# Supplementary material for: A Simplified and Efficient Method for Production of Manganese Ferrite Magnetic Nanoparticles and Their Application in DNA Isolation
Source: Int J Mol Sci. 2023 Jan 21;24(3):2156. doi: 10.3390/ijms24032156 (PMC9917137; doi:10.3390/ijms24032156)
Supplement: Supplementary file 1 [file ijms-24-02156-s001.zip › ijms-1957204-supplementary.pdf]

A simplified and efficient method for production of manganese ferrite magnetic nanoparticles and their application in DNA isolation

Tímea Beatrix Gerzsenyi [1], Ágnes Mária Ilosvai [2], Gergely Szilágyi [2], Milán Szőri [2], Csaba Váradi [1], Béla Viskolcz [1,2], László Vanyorek [2,*] and Emma Szőri-Dorogházi [1,*]

[1]Higher Education and Industrial Cooperation Centre, University of Miskolc, 3515 Miskolc, Hungary
[2]Institute of Chemistry, Faculty of Materials and Chemical Engineering, University of Miskolc, 3515 Miskolc, Hungary
*Correspondence: kemvanyi@uni-miskolc.hu, emma.szdoroghazi@uni-miskolc.hu

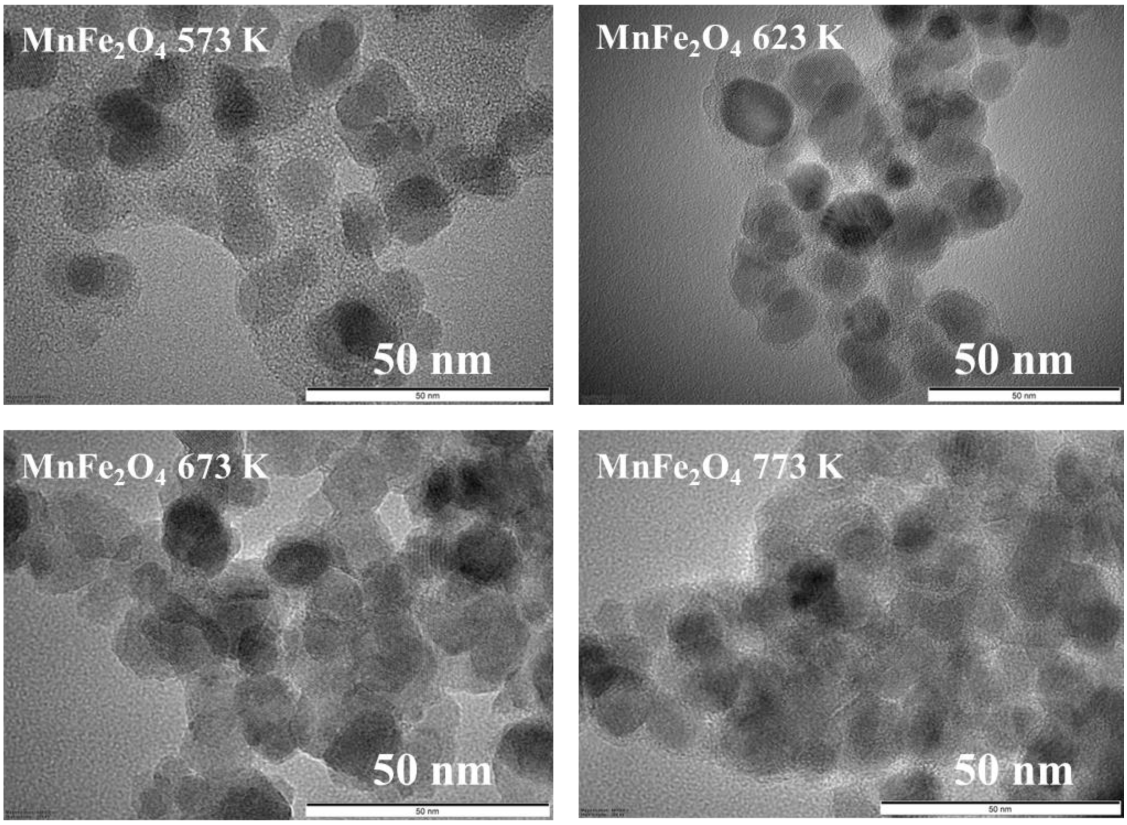

Figure S1. HRTEM images of the manganese ferrite samples synthetised at 573K, 623K, 673K, and 773K.

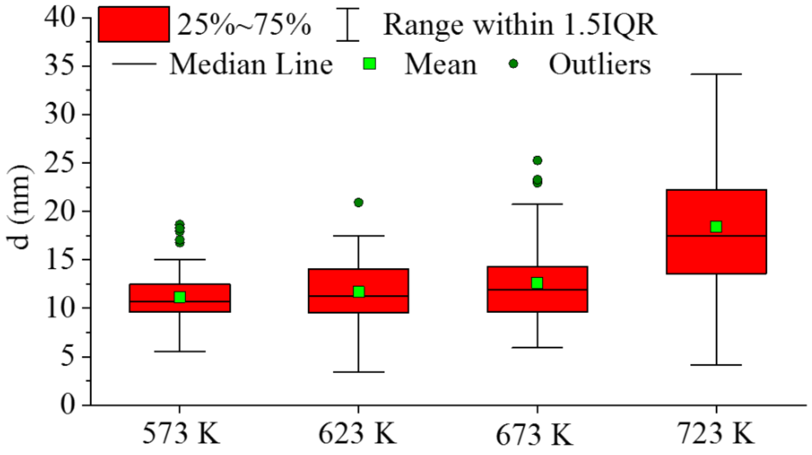

Figure S2. Box plot diagrams of the particle sizes in the manganese ferrite magnetic nanoparticles synthetied at 573K, 623K, 673K, and 773K. The particle sizes were obtained from the analysis of TEM images.

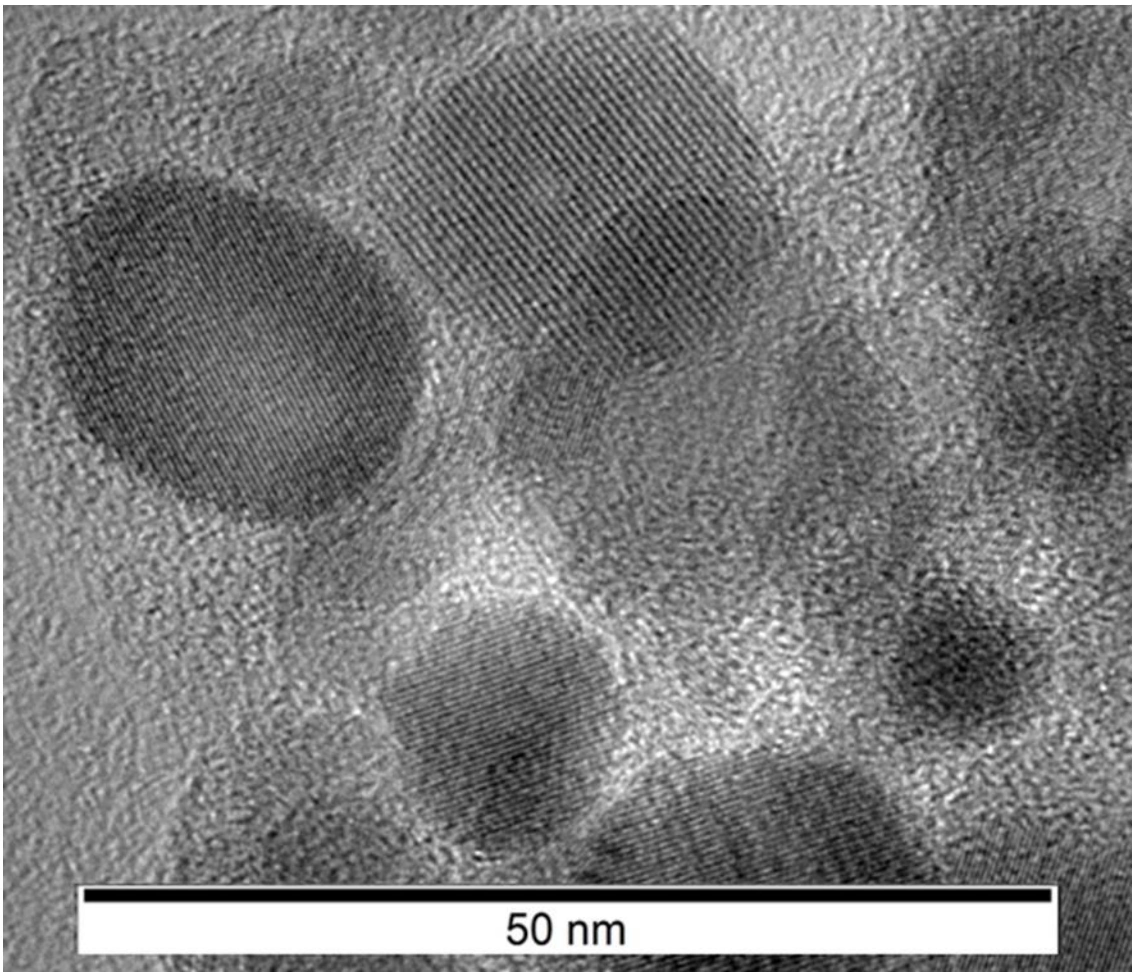

Figure S3. HRTEM picture of the $MnFe_2O_4$ sample.

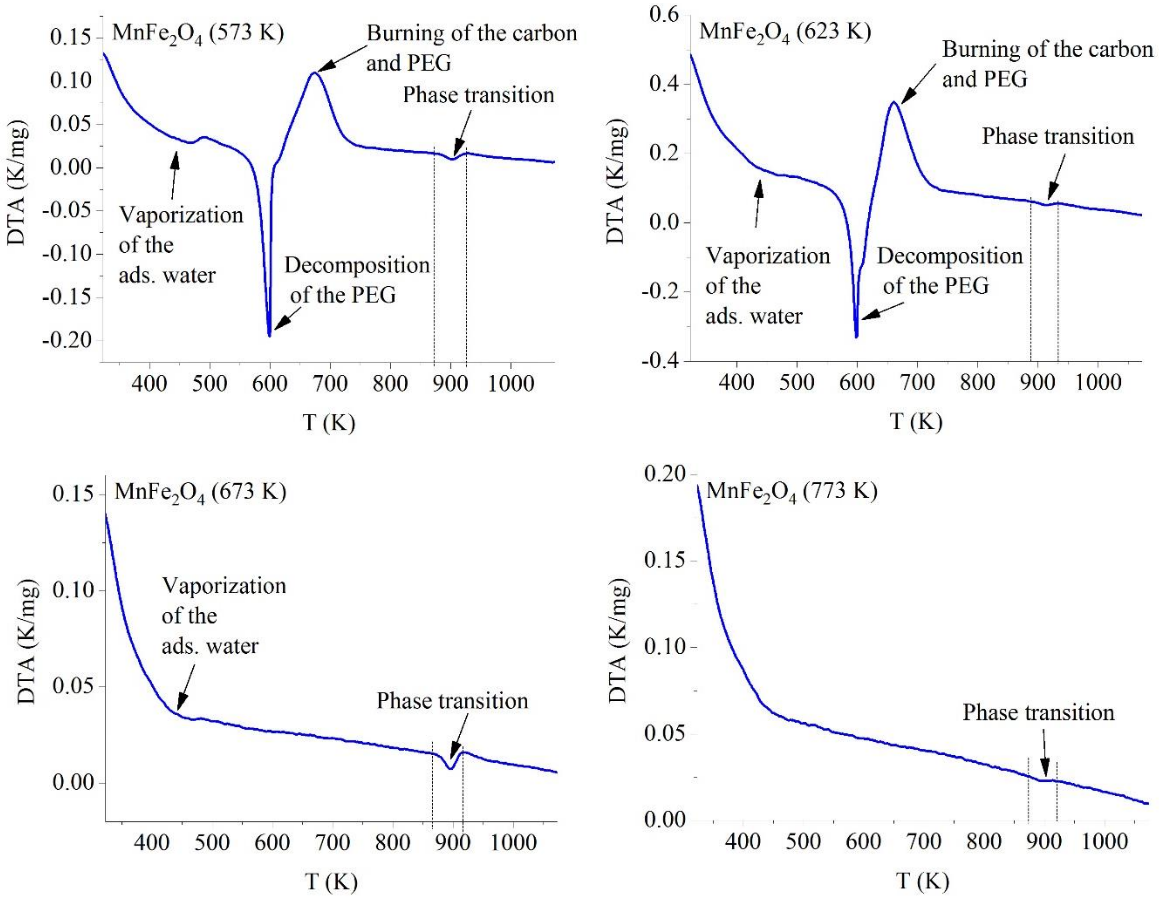

Figure S4. TDA curves of the manganese ferrite samples.

Table S1. Comparison of the coercivity, saturation magnetization, crystallite and particle size of the different ferrite nanoparticles.

Formula of ferrite	Coercivity Hc (Oe)	Ms (emu/g)	Crystallite size by XRD (nm)	Particle size by TEM (nm)	References
**$MnFe_2O_4$ (573K)**	0.7	72.0	11.0	11 ± 3	Present work
**$MnFe_2O_4$ (623K)**	-	-	11.0	12 ± 3	
**$MnFe_2O_4$ (673K)**	-	-	13.0	13 ± 4	
**$MnFe_2O_4$ (773K)**	-	-	14.0	18 ± 6	
**$MnFe_2O_4$**	94.1	15.9	16.1	-	https://doi.org/10.1016/j.matpr.2021.01.209
	95.2	14.3	14.4	-	
	93.7	14.8	11.5	-	
	40.0	41.0	8.4	24	https://doi.org/10.1016/j.ceramint.2014.11.066
	~ 0	66.4	12.0	12	https://doi.org/10.1016/j.jmmm.2016.10.105
	~ 0	60.8	9.7	9	
**$NiFe_2O_4$**	93.4	67.8	29.0	29	https://doi.org/10.1016/j.matchemphys.2022.126793
	94.9	0.8	20.0	24	https://doi.org/10.1016/j.physb.2022.414232
	0.6	35.1	13.0	10-15	https://doi.org/10.1186/1752-153X-6-23.
	0.6	34.5	12.0	10-15	
	15.7	39.6	53.0	60	
**$CoFe_2O_4$**	508.5	84.8	-	25	https://doi.org/10.1016/j.jmmm.2022.170073
	207.0	49.2	8.7	11 ± 5	https://doi.org/10.1016/j.ceramint.2022.06.104
	188.1	33.6	5.7	5 ± 1	https://doi.org/10.1016/j.jallcom.2020.155710
	44.0	39.6	8.0	8 ± 1	
	19.30	56.7	9.1	9 ± 1	
	556.6	21.7	-	20-160	https://doi.org/10.1016/j.csite.2021.101040
**$MgFe_2O_4$**	~ 0	8.5	8.80	8	https://doi.org/10.1016/j.jmmm.2016.08.057
	8.5	12.8	11.1	13	https://doi.org/10.1016/j.jpcs.2021.110051
	9.9	18.6	15.1	16	
	22.3	24.5	22.3	24	
	59.7	29.4	32.7	33	
	419.0	31.8	24.0	15-35	https://doi.org/10.1016/j.mseb.2016.05.019
	106.0	23.9	24.0		
	418.5	0.4	25.1	20-30	https://doi.org/10.1016/j.physb.2020.412660
**$ZnFe_2O_4$**	94.9	0.2	16.0	21.00	https://doi.org/10.1016/j.physb.2022.414232
	~ 0	41.5	25.0	310	https://doi.org/10.1016/j.physb.2020.412015
	90.0	77.0	80.0	375	
	9.9	3.1	19.0	-	https://doi.org/10.1016/j.matpr.2022.08.032
	14.5	19.4	41.1	-	
